# Indigestible Foreign Bodies in the Rumen and Reticulum of Cattle Slaughtered at ELFORA Abattoir, Kombolcha Town, Amhara Region, Ethiopia

**DOI:** 10.1155/vmi/7342530

**Published:** 2026-05-08

**Authors:** Teshager Dubie, Sahle Demse Killu, Ashenafi Syoum, Fanuel Bizuayehu Yihunie

**Affiliations:** ^1^ Department of Veterinary Medicine, College of Veterinary Medicine and Animal Sciences, Samara University, P.O. Box 132, Samara, Ethiopia, su.edu.et

**Keywords:** abattoir, cattle, foreign body, Kombolcha, occurrence, reticulum, rumen

## Abstract

Any nondigestible material ingested by an animal that cannot be broken down by digestive enzymes or rumen microorganisms and remains within the gastrointestinal tract, potentially causing mechanical or functional disturbances, is referred to as an indigestible foreign body. This study aims to evaluate the occurrence of indigestible foreign bodies in the rumen and reticulum of cattle slaughtered at the Kombolcha ELFORA Abattoir, identify the types of foreign bodies, and assess the associated risk factors that contribute to their ingestion. A cross‐sectional study was conducted from November 2024 to June 2025 at the Kombolcha ELFORA Abattoir, Ethiopia. Postmortem examinations were employed to assess the animals and recover foreign bodies, respectively, from the rumen and reticulum of cattle slaughtered at the abattoir. The study animals were selected using a simple random sampling technique from the total cattle slaughtered during the study period. Data analysis included descriptive statistics and Pearson’s chi‐square (*χ* ^2^) test to check associations between the prevalence of indigestible foreign bodies and various potential risk factors. In this study, the overall occurrence of foreign bodies in slaughtered cattle was found to be 35.3% (*n* = 127/360). A higher prevalence was recorded in females (42%), crossbred cattle (36.36%), older animals (45.7%), and cattle with poor body condition scores (38.1%). Among the assessed associated factors, age (*p* = 0.025) and body condition score (*p* = 0.022) showed a statistically significant association with the occurrence of foreign bodies. Plastics were the most frequently encountered materials in the rumen, while the reticulum commonly retained metallic objects. The most commonly recovered nonpenetrating materials included plastic bags (19.68%), sand (18.1%), cloth (8.66%), rope (7.87%), and leather (3.87%). Penetrating foreign bodies primarily consisted of wire (6.29%) and nails (5.51%). Plastic bags were the most commonly recovered foreign materials, followed by sand, cloth, rope, and wire. The findings of this study highlight that the ingestion of both metallic and nonmetallic foreign bodies is a prevalent and significant health concern in cattle slaughtered at the abattoir. Implementing effective solid waste management practices is critically important for safeguarding cattle health in the study area.

## 1. Introduction

Ethiopia possesses Africa’s largest livestock population, estimated at about 70 million cattle, 42 million sheep, 52 million goats, 8 million camels, 13.33 million equines, and 56 million chickens [1, 2]. The country’s diverse agro‐ecological zones and favorable environmental conditions support a wide range of livestock species and make it well suited for livestock production [3]. Despite its considerable potential, the benefits of indigenous livestock resources have not been fully realized because of various challenges hindering sectoral development [4]. Major causes of livestock losses include endemic diseases, recurrent drought, inadequate veterinary services, and limited government support [5]. Cattle are vital to the Ethiopian economy as major sources of meat, milk, and household income. However, their productivity is constrained by poor nutrition, animal diseases, limited support services, and foreign exchange challenges. Consequently, as with other livestock species, their contribution remains below potential due to the high burden of disease, inadequate management practices, and low genetic performance [3].

Ingestion of indigestible foreign materials by cattle and buffaloes is a widespread global problem known as foreign body syndrome (FBS) [6]. Cattle are more prone to foreign body ingestion than small ruminants because of their indiscriminate feeding behavior and limited oral discrimination, as they do not use their lips effectively for prehension and their hard palate has poor tactile sensitivity to foreign objects [7]. Furthermore, cattle with mineral deficiencies are more likely to ingest objects with a mineral or metallic taste. These indigestible foreign bodies (IFBs) are classified into two categories: metallic (nails, wires, needles, screws, coins, pins, and razor blades) and nonmetallic (plastic bags, cloth, rope, leather, rubber, sand/stones, paper, and polythene) origin. The typical foreign body is a metallic object, such as a piece of wire or a nail, often greater than 2.5 cm in length [8, 9].

Foreign body ingestion in cattle is a significant cause of morbidity and may result in death. It is mainly associated with nutritional deficiencies and poor feeding management and primarily affects the rumen and reticulum. Clinical manifestations depend on the duration of retention, anatomical location, degree of obstruction, and the physical nature of the ingested material [10]. Nonmetallic foreign bodies within the reticulorumen are frequently responsible for recurrent ruminal tympany in adult dairy cattle and, over time, may form large compact masses that lead to anorexia, decreased productivity, and progressive loss of body condition [11].

The presence of foreign bodies in the rumen and reticulum interferes with the absorption of volatile fatty acids (VFAs), thereby reducing growth performance and fattening efficiency. Perforation of the reticular wall permits leakage of ingesta and bacteria into the peritoneal cavity, resulting in localized or diffuse peritonitis. In some cases, swallowed objects may penetrate the pleural cavity, causing pleuritis and pneumonitis, or extend into the pericardial sac, leading to pericarditis [12]. This condition is particularly serious in urban and periurban areas where extensive construction activities and improper disposal of plastic and metallic wastes expose dairy cattle to foreign body ingestion [13]. In Ethiopia, information regarding the magnitude and occurrence of forestomach foreign bodies remains limited. Moreover, ruminal impaction caused by these materials is often asymptomatic and is usually detected only when large accumulations occur; therefore, abattoir‐based studies provide an important opportunity for adequate assessment of the problem [14].

The types and prevalence of foreign bodies in the rumen and reticulum of cattle have been previously reported by Mohammed et al. [15] at the Badano Woreda Municipal Abattoir in eastern Ethiopia, Kassahun et al. [16] at the Nekemte Municipal Abattoir in western Ethiopia, and Sheferaw et al. [17] at the Gondar ELFORA Abattoir in northwestern Ethiopia. Despite the recognized importance of this condition, recent studies examining the types, prevalence, and associated risk factors of foreign body ingestion in cattle, particularly in the selected areas of origin, are limited. Therefore, the present study aims to determine the occurrence of foreign bodies in the rumen and reticulum of cattle slaughtered at the Kombolcha ELFORA Abattoir, identify the types of foreign materials encountered, and assess the associated risk factors contributing to their ingestion in the study areas.

## 2. Materials and Methods

### 2.1. Description of the Study Area, Study Population, and Period

This cross‐sectional study was conducted at the Kombolcha ELFORA Abattoir in South Wollo Zone, Ethiopia, from November 2024 to June 2025 on apparently healthy slaughtered cattle. Kombolcha is located about 375 km northeast of Addis Ababa at an altitude of 1500–1840 m above sea level, with an annual rainfall of 750–900 mm, mean temperatures of 11.7°C–23.9°C, bimodal rainfall (March–May and June–September), and relative humidity ranging from 23.9% to 79% [18]. Both local and crossbred cattle originating from Kemise, Chefa, Harbu, Kombolcha, Dessie, Tincha, Kutaber, and Haik were included and classified by age as young (≤ 5 years), adult (5–10 years), and old (≥ 10 years) based on dentition [19]. The body condition of the study animals was assessed using a standardized scoring system ranging from 0 to 5 [20], where score 0 indicates the poorest condition and score 5 the best. For analysis, cattle were categorized into three groups: poor (scores 0–1), medium (scores 2–3), and good (scores 4–5). Breed, age, sex, body condition score, and origin were assessed as risk factors to determine the prevalence and types of IFBs in cattle.

### 2.2. Sampling Technique and Sample Size Determination

A simple random sampling technique was employed to select the study animals, and the rumen and reticulum of individual animals were examined. The sample size was determined according to Thursfield [21] using a previous prevalence study (37.5%) by Kebede et al. [22], a 95% confidence interval, and a 0.05 desired absolute precision. This is calculated by using the following formula:

(1) N=1.962xPexp1−Pexpd2,

where

*N* = required sample size.

*P*_exp_ = expected prevalence (0.375).

D = eesired absolute precision (0.05).

Therefore, the number of cattle examined in this study was 360.

### 2.3. Study Methodology

#### 2.3.1. Postmortem Examination

Postmortem examination involved visual inspection, palpation, and making incision of the rumen and reticulum to look for the presence of IFBs in the rumen and reticulum. All the contents were examined thoroughly for the presence of foreign bodies. Any foreign bodies obtained during inspection were washed with water to remove adhering feed material and identify the type of foreign bodies. When the finding was positive, the location and type of the foreign bodies were recorded; otherwise, they were recorded as negative in the postmortem record sheet. In addition to these, gross lesions associated with these foreign bodies were documented.

### 2.4. Data Management and Analysis

The collected data were entered into Microsoft Excel and analyzed using SPSS Version 27. Descriptive statistics were employed to summarize the data. The prevalence of IFBs was determined by dividing the number of positive cases by the total number of animals examined. The Pearson chi‐square (*x* ^2^) test was employed to check associations between the prevalence of IFB and potential risk factors. A *p* value less than 0.05 was considered statistically significant.

## 3. Results

### 3.1. Demographic Characteristics of the Study Animals

Descriptive statistics showed that 75.3% of the cattle were males, 63.3% were adults (5–10 years), and 75.6% were local breeds. In addition, 53.9% had poor body condition, and 35.3% originated from the Chefa area (Table 1).

**TABLE 1:** Demographic characteristics of cattle examined at the study area (*n* = 360).

Variable	Category	Frequency	Percentage
Sex	Male	272	75.6
	Female	88	24.4

Age	Young	27	7.5
	Adult	228	63.3
	Old	105	29.2

Breed	Local	272	75.6
	Cross	88	24.4

BCS	Good	27	7.5
	Medium	139	38.6
	Poor	194	53.9

Origin	Kemise	33	9.2
	Chefa	127	35.3
	Harbu	27	7.5
	Kombolcha	17	4.7
	Dessie	67	18.6
	Tincha	18	5.0
	Kutaber	36	10.0
	Haik	35	9.7

### 3.2. Prevalence of Foreign Bodies in Cattle

Out of the 360 cattle examined for the presence of foreign bodies in the rumen and reticulum, 127 animals (35.3%) tested positive. Among these, 55 cases (43.3%) involved foreign bodies located in the rumen, 41 cases (32.3%) in the reticulum, and 31 cases (24.4%) had foreign bodies in both compartments. Various types of indigestible materials were identified, including combinations such as sand and plastic bags, rope and sand, plastic bags and wire, stones and plastic bags, rope and wire, cloth and wire, as well as individual materials like plastic bags, cloth, sand, nails, wire, hair, rope, stone, and leather.

#### 3.2.1. Prevalence of Foreign Bodies Based on the Origin of the Cattle

The study animals slaughtered at the Kombolcha ELFORA Abattoir during this study originated from various agro‐ecological zones, including Kemise, Chefa, Harbu, Kombolcha, Dessie, Tincha, Kutaber, and Haik. The highest prevalence of foreign bodies was recorded in cattle originating from Kombolcha, while the lowest was observed in those brought from Kemise. However, statistical analysis showed no significant difference in the occurrence of foreign bodies among cattle from the various locations (*p* = 0.919) (Table 2).

**TABLE 2:** Prevalence of foreign body ingestion among cattle categorized according to their geographical or farm of origin (*n* = 360).

Origin	Number of examined animals	Number of positive animal	Prevalence (%)	Chi‐square (*x* ^2^)	*p* value
Kemise	33	9	27.27	2.607	0.919
Chefa	127	44	34.6		
Harbu	27	10	37		
Kombolcha	17	7	41		
Dessie	67	26	38.8		
Tincha	18	7	38.8		
Kutaber	36	14	38.8		
Haik	35	10	28.6		
Total	360	127			

#### 3.2.2. Prevalence of Foreign Bodies in Cattle by Sex, Age, Body Condition Score, and Breed

Among the 360 cattle examined, IFBs were detected in the rumen and reticulum of 90 male (33.1%) and 37 female cattle (42%). Although the prevalence was higher in females, the difference between male and female cattle was not statistically significant (*p* = 0.244), whereas older animals showed the highest prevalence of IFBs compared to the younger age groups. Statistical analysis confirmed a significant association between age and foreign body occurrence (*p* = 0.025), indicating that age is a potential risk factor. Furthermore, the prevalence of IFBs varied with body condition scores. Specifically, three cattle (11.1%) with good body condition, 50 (35.97%) with medium condition, and 74 (38.1%) with poor condition were tested positive for foreign bodies. The highest prevalence was observed in cattle with poor body condition, followed by those with medium condition. Statistical analysis revealed a significant association (*p* = 0.022) between body condition score and the presence of foreign bodies in the rumen and reticulum. Moreover, the prevalence of rumen and reticulum foreign bodies was higher in crossbred cattle compared to local breeds. However, the difference in occurrence between the two breed types was not statistically significant (*P* = 0.060) (Table 3).

**TABLE 3:** Prevalence of foreign bodies in cattle according to sex, age, BCS, and breed (*n* = 360).

Variables	Category	Number of animals examined	Prevalence (%)	Chi‐square (*x* ^2^)	*p* value
Sex	Male	272	33.1	2.818	0.244
	Female	88	42		

Age	Young	27	25.9	7.409	0.025
	Adult	228	31.57		
	Old	105	45.7		

BCS	Good	27	11.1	7.634	0.022
	Medium	139	35.97		
	Poor	194	38.1		

Breed	Local	272	34.9	0.060	0.806
	Cross	88	36.36		

### 3.3. Prevalence of Foreign Bodies Based on Their Types

**FIGURE 1:**
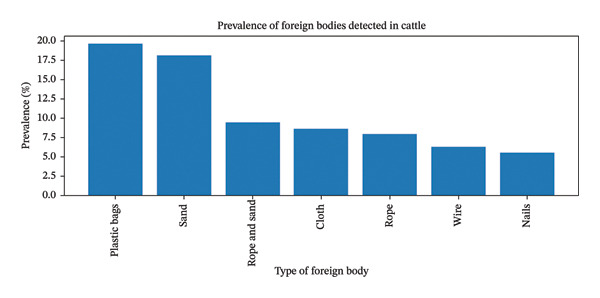
Types of foreign body detected in cattle at the Kombolcha ELFORA Abattoir.

Various foreign bodies were identified in the rumen and reticulum of slaughtered cattle, with prevalence ranging from 0.79% to 19.68%. Plastic bags were the most common (19.68%), followed by sand (18.1%), rope and sand (9.4%), cloth (8.66%), rope (7.87%), wire (6.29%), and nails (5.51%), indicating that plastic bags and sand were the predominant indigestible materials ingested by cattle (Figure 1).

### 3.4. Prevalence of Foreign Bodies Based on the Location of the Compartment

Out of the 360 cattle examined for foreign bodies in the rumen and reticulum, 127 animals (35.3%) were found positive. Among these cases, 43.3% of the foreign bodies were located in the rumen, 32.3% in the reticulum, and 24.4% in both compartments. The rumen showed a higher proportion of foreign body lodgment than the reticulum. Statistical analysis demonstrated a highly significant association between the anatomical location of foreign bodies and their prevalence (*P* = 0.001), indicating that the distribution of foreign bodies varied significantly between the rumen and reticulum (Table 4).

**TABLE 4:** Prevalence of foreign bodies by the compartment (location).

Location	No. of animal positive	Prevalence (%)	Chi‐square	*p* value
Rumen	55	43.3	347.017	0.001
Reticulum	41	32.3		
Both	31	24.4		
Total	127	100		

## 4. Discussion

The present study revealed an overall prevalence of foreign bodies of 35.3% (*n* = 127) in the rumen and reticulum of cattle slaughtered at the Kombolcha ELFORA Abattoir. The current prevalence of foreign bodies observed in this study closely aligns with the findings of Megersa [23], who reported a 35.4% prevalence in cattle slaughtered at Beko Town slaughterhouse in Fedi District. It is slightly lower than the 37.75% prevalence reported by Shimels et al. [24] at the Kombolcha ELFORA Abattoir. Additionally, Amin and Fentahun [25], who examined 384 cattle at Haramaya and Awaday municipal abattoirs between November 2017 and March 2018, reported a higher prevalence of 41.7%. Similarly, the prevalence observed in the present study is significantly lower than the 77.41% reported by Ismael et al. [26] in adult dairy cattle suffering from recurrent rumen tympany due to IFBs in Jordan. Additionally, Jagos [27] reported a higher overall prevalence of 51% in adult cows, with 63% of foreign bodies located in the rumen and 15% in the reticulum. However, the current study’s prevalence is higher than the 13.4% reported by Daba Gudata [28] in cattle slaughtered at the Gimbi Municipal Abattoir and also exceeds the 30.68% prevalence reported by Dressa et al. [29] at the Bishoftu ELFORA Export Abattoir. The variation in prevalence across studies may be attributed to differences in waste management practices among the study areas. Additionally, the timing of each study could influence the results. In recent years, there has been a growing trend toward the intensification of animal management systems, which may reduce animals’ exposure to foreign materials, as they are increasingly confined within controlled environments for extended periods.

The highest occurrence of foreign bodies was observed in cattle originating from Kombolcha (41%), followed by Dessie, Kutaber, and Tincha, each with a prevalence of 38.8%. According to Jebessa et al. [30], Ismael et al. [26], Misk et al. [9], and Singh and Nigam [31], variations in prevalence across locations may be attributed to differences in animal origin and community awareness regarding waste management. In towns like Kombolcha, the presence of construction sites, improper disposal of plastic bags from shops and markets, and general environmental pollution increases the likelihood of foreign body ingestion. Additionally, factors such as industrialization, mechanization of agriculture, and limited grazing areas force animals to forage near towns, roadsides, and marketplaces, areas often contaminated with indigestible materials.

In the present study, a higher prevalence of foreign bodies was observed in female cattle (42%) compared to males (33.1%). This finding is consistent with the results reported by Mohamed Asledin [32] in selected districts of East Hararghe Municipal Abattoirs and Aragaw et al. [33] at the Awassa Municipal Abattoir, both of which indicated a higher occurrence of foreign bodies in female cattle. This increased prevalence in females may be linked to physiological stressors such as lactation and pregnancy, which elevate nutritional demands and appetite, potentially leading to indiscriminate ingestion of foreign materials. The higher prevalence of foreign bodies was observed in older cattle (45.7%) compared to adults (31.57%) and young animals (25.9%). This finding aligns with the results reported by Megersa Mussa [34], who recorded prevalence rates of 50% in older, 30% in adult, and 21.4% in young cattle, as well as by Anteneh [35], who reported 25.6%, 13.5%, and 4.95% in the respective age groups. The increased prevalence in older animals may be attributed to cumulative exposure, leading to greater chances of ingesting and retaining indigestible foreign materials.

In this study, the highest occurrence of rumen and reticulum foreign bodies was observed in cattle with poor body condition (38.1%), followed by those with medium (35.97%) and good body condition (11.1%). These findings align with the reports of Bewuketu Anteneh (2015), who recorded prevalence rates of 39.72% in poor, 11.4% in medium, and 8.7% in good body condition animals. Similarly, Desiye and Mersha [14] reported a higher frequency of foreign body detection in poorly conditioned cattle. Poor body condition may result from the presence of foreign bodies, which can interfere with nutrient absorption, particularly of VFAs leading to weight loss and reduced productivity.

In the current study, the prevalence of foreign bodies was slightly higher in crossbred cattle (36.36%) compared to local breeds (34.9%). This result is consistent with findings by Desiye and Mersha [14], who reported a prevalence of 70% in crossbreeds and 10.77% in local breeds at the Jimma Municipal Abattoir, as well as Ebsa Amin and Tewodros Fentahun [36], who documented a higher prevalence of forestomach foreign bodies in crossbred cattle (75%) than in local breeds (38.6%) at Haramaya and Awaday municipal abattoirs. The increased prevalence in crossbreeds may be attributed to their higher nutritional demands due to greater productivity, which may lead to indiscriminate feeding behavior and, consequently, an increased risk of ingesting foreign bodies.

Plastic bags were the most frequently identified foreign bodies in this study, accounting for 19.68%, followed by sand (18.1%), cloth (8.66%), and rope (7.86%). These findings are in line with the results of Ebsa Amin and Tewodros Fentahun [36], who reported plastics (41.20%) as the most common foreign material, followed by cloth (22.05%) and rope (17.65%) in cattle slaughtered at Haramaya and Awaday municipal abattoirs. Similarly, Bewuketu Anteneh [35] found plastics (47.3%) to be the most prevalent foreign bodies at the Gondar ELFORA Abattoir, followed by rope and cloth. The high occurrence of plastic ingestion may be attributed to poor waste disposal practices and feed scarcity, which are a significant challenge in the current study area. It has been suggested that prolonged feed shortages during dry seasons increase the likelihood of plastic ingestion, often driven by deficiencies in essential nutrients such as minerals and vitamins.

In this study, of the 35.3% of cattle that tested positive for foreign bodies, 43.3% were located in the rumen, 32.3% in the reticulum, and 24.4% in both compartments. The prevalence was notably higher in the rumen compared to the reticulum. This finding is consistent with the results of Amin and Fentahun [25], who reported a significantly higher prevalence of foreign bodies in the rumen (80.6%) than in the reticulum (8.8%) in cattle slaughtered at Haramaya and Awaday municipal abattoirs. The greater occurrence in the rumen is likely due to its larger capacity and its role as the primary site where ingested materials, especially low‐density items, settle and accumulate.

## 5. Conclusion and Recommendations

Foreign body ingestion is a common problem in cattle, causing high morbidity, mortality, and reduced productivity, especially in developing countries with poor management practices. Cattle in poor body condition, crossbreeds, and those ≥ 10 years old are most affected by IFBs. The study identified plastic bags, sand, rope, cloth, leather, nails, and wire as common foreign bodies, with plastic bags being the most frequent. Nonmetallic objects were mainly found in the rumen, while metallic ones were lodged in the reticulum. Preventive measures include restricting cattle access to construction sites and unclean grazing areas, educating owners, and promoting public awareness to reduce environmental waste.

### 5.1. Limitation of the Study

This study was limited by its cross‐sectional design, as a single‐time survey cannot account for seasonal variation in foreign body ingestion related to feed scarcity or grazing conditions. Slaughtered cattle may not fully represent the general population, introducing potential selection bias. Additionally, small or partially digested foreign bodies may have been missed during inspection, and foreign bodies were not weighed during the postmortem examination, which represents another limitation of the study.

## Funding

This research work did not receive any financial support from any institutions or individuals.

## Ethics Statement

All study participants were informed about the purpose of the study, and verbal consent was obtained prior to their participation. Verbal consent was deemed sufficient because the study involved interviews only, and no procedures posed any risk to the participants, provided their privacy remained protected. All respondents were adults aged 18 years and above. Confidentiality of the data and scientific integrity were strictly maintained throughout the study.

## Conflicts of Interest

The authors declare no conflicts of interest.

## Data Availability

The data that support the findings of this study are available from the corresponding author upon reasonable request.
